# Van Wyk-Grumbach syndrome in a female pediatric patient with trisomy 21: a case report

**DOI:** 10.1186/s13633-020-0072-y

**Published:** 2020-01-28

**Authors:** Jyotsna Gupta, Karen Lin-Su

**Affiliations:** Division of Pediatric Endocrinology, Department of Pediatrics, Weill Cornell Medicine, New York Presbyterian Hospital, 505 East 70th Street, New York, NY NY-10021 USA

**Keywords:** Hypothyroidism, Vaginal bleeding, Precocious puberty, Van Wyk-Grumbach, Trisomy 21

## Abstract

**Background:**

Children with hypothyroidism typically present with delayed growth and development, but on rare occasions can present with signs of precocious puberty. This presentation is called Van Wyk-Grumbach syndrome. Van Wyk-Grumbach syndrome has seldom been described in patients with trisomy 21.

**Case presentation:**

We present the case of a 4-year-old girl with trisomy 21, who recently moved to the United States from Guyana, and presented to the emergency room with recurrent vaginal bleeding. She was eventually diagnosed with hypothyroidism and Van Wyk-Grumbach syndrome. She was noted to have Tanner I breasts and pubic hair. A pelvic ultrasound was performed, which showed a simple cyst in the right adnexa. Subsequent laboratory evaluation revealed a thyroid stimulating hormone (TSH) of > 150 mIU/ml along with low free thyroxine of 0.3 ng/dl, suggesting longstanding untreated hypothyroidism. Estradiol and alpha-fetoprotein (AFP) levels were elevated. Bone age was delayed. The patient was diagnosed with Van Wyk-Grumbach syndrome and was started on levothyroxine therapy with subsequent resolution of vaginal bleeding. Estradiol and AFP both normalized after initiating levothyroxine therapy.

**Conclusion:**

This case emphasizes the importance of recognizing the presence of precocious puberty, delayed bone age and ovarian cyst as a manifestation of primary hypothyroidism. In addition, it highlights the need for thyroid function screening in patients with Trisomy 21. Tumor markers may be elevated in Van Wyk-Grumbach syndrome with subsequent normalization after treatment.

## Background

Children with hypothyroidism typically present with delayed growth and development, but on rare occasions can present with signs of precocious puberty. Boys may present with enlargement of testicles and girls may present with menarche, with or without the development of breasts. This presentation was described by Van Wyk and Grumbach in 1960 and is called Van Wyk-Grumbach syndrome. The etiology has been thought to be related to high levels of TSH acting on FSH receptors due to molecular similarities between the glycoprotein receptors of these two hormones, which share a common subunit [1].

## Case presentation

A 4-year-old girl with trisomy 21, who had recently moved to the United States from Guyana, presented to the emergency room with a one-week history of vaginal bleeding. Her mother reported four prior episodes of recurrent bleeding in Guyana over the past year. Review of her prior medical records from Guyana included a pelvic ultrasound that showed a right ovarian cyst measuring 4.1 cm × 2.3 cm. Laboratory evaluation was negative for central precocious puberty or congenital adrenal hyperplasia. Thyroid function tests were not obtained. When she presented to the emergency room, a repeat pelvic ultrasound showed a 3.7 × 3.4 cm simple cyst in the right adnexa.

She was sent to our pediatric endocrinology clinic for further evaluation. She was born in Guyana and was the product of a full-term pregnancy. Her birth weight was 2.28 kg, and she was diagnosed with trisomy 21 a few weeks after birth based on genetic testing. On exam, her height was 90 cm [8th percentile on Down Syndrome curve [2]; < 1st percentile on CDC growth curve [3], weight was 13.7 kg (19th percentile on Down Syndrome curve; 2nd percentile on CDC growth curve), and BMI was 16.9 kg/m2 (87th percentile). Vital signs were normal for age. She had low energy and hypotonia. She had facial features consistent with trisomy 21. On pubertal exam, she was noted to have Tanner I breasts and pubic hair. She had no axillary hair or body odor. A small amount of fresh blood was noted in the vaginal orifice; otherwise, her external genitalia were unremarkable.

Her initial labs were notable for normal platelet count, mild microcytic anemia, markedly elevated TSH of > 150 mIU/mL with a low free thyroxine level of 0.3 ng/dL and a low total thyroxine level of < 0.3 mcg/dL with an elevated thyroid peroxidase antibody level of 1763 IU/mL and elevated thyroglobulin antibody level of 85.2 IU/mL, indicating Hashimoto’s thyroiditis. Her LH level was prepubertal at < 0.02 mIU/mL. FSH level was 12.64 mIU/mLand estradiol levels were elevated at 154.0 pg/mL. The lab at our institution utilizes estradiol extraction method and liquid chromatography mass spectrometry for analysis of LH and FSH. Interestingly, her AFP level was more than three times the upper limit of normal at 31.0 ng/mL (Table 1).

**Table 1:** Review of laboratory evaluation over the clinical course of treatment with Levothyroxine

Laboratory parameter	Normal range	At time of diagnosis	1 month after treatment	2 months after treatment	9 months after treatment
TSH	0.54–4.53 mIU/mL	> 150	43.5	9.2	1.3
Free T4	0.85–1.75 ng/dL	0.3	1.2	1.6	1.4
Total T4	7.3–15.0 mcg/dL	< 0.3			9.4
TPO antibodies	0.0–9.0 IU/mL	1763.0			
Tg antibodies	0.0–4.0 IU/mL	85.2			
LH	3–7 years: < or = 0.26 mIU/mL	< 0.02			
FSH	0–4 years: Not established 5–9 years: 0.72–5.33 mIU/mL	12.64			
Estradiol, 17 beta	Tanner I < 25 pg/mL	154.0	18.6	< 1.0	
Testosterone total	Prepubertal Females: < 2.5–10 ng/ dL	< 2.5			
AFP	<=8.1 ng/mL	31.0	12.2	2.9	

*TSH* thyroid stimulating hormone, *T4* thyroxine, *TPO* thyroid Peroxidase, *Tg* thyroglobulin, *LH* luteinizing hormone, *FSH* follicle stimulating hormone, *AFP* alpha-fetoprotein

A clinical diagnosis of Van Wyk-Grumbach syndrome was made, and she was started on levothyroxine at a dose of 50 micrograms per day (4.1 mcg/kg/day). Her TSH level improved to 9.2 mIU/mL and her free thyroxine level normalized to 1.6 ng/dL within 2 months of initiating treatment. Her AFP level normalized to 2.9 ng/mL on repeat testing 2 months later as well (Table 1). A bone age, which was subsequently obtained at chronological age of 5 years 5 months, was significantly delayed with a reading of 2 years. At her follow up visit, her mother reported resolution of vaginal bleeding and remarkable improvement in her energy level and school performance. Repeat pelvic ultrasound obtained 10 months after treatment initiation showed that the cyst had resolved.

## Discussion and conclusions

Van Wyk-Grumbach syndrome was first described in 1960 [4] as a syndrome of juvenile hypothyroidism associated with pubertal advancement. It is a rare clinical entity that presents with prolonged untreated hypothyroidism. Girls with Van Wyck-Grumbach syndrome [5] can have varying degrees of pubertal development as well as multicystic ovaries, vaginal bleeding, galactorrhea and delayed bone age. Boys usually present with testicular enlargement without virilization. The etiology of Van Wyck-Grumbach syndrome is thought to be related to complex interactions within the hypothalamic-pituitary axis. In a study by Anasti et al. [6], recombinant TSH interacted with FSH receptor to stimulate adenylyl cyclase activity. Recombinant TSH acted as a competitive inhibitor of FSH, indicating that TSH and FSH were acting through the FSH receptor. Increased FSH levels can result in ovarian hyperstimulation; hence, multicystic ovaries can be a feature of this syndrome as well [7]. Ovarian cysts may resolve after levothyroxine treatment, as was demonstrated in our patient.

Another interesting finding in our patient was the presence of an elevated AFP level. An AFP level was obtained in our patient due to the presence of an ovarian mass and concern for possible malignancy. Elevated AFP levels have been reported previously in patients with hypothyroidism [8]. Elevation of tumor markers [such as AFP [9], CA-125 [10], LDH, and Inhibin] has also been described in other case reports of Van Wyk-Grumbach syndrome as well. An elevated tumor marker can cause significant anxiety for both patients as well as clinicians. For our patient, the AFP levels normalized within 2 months of treatment. Therefore, it is important to recognize that elevated tumor markers should be repeated during the course of treatment, and further imaging with an MRI may not be necessary.

Delayed bone age can further help point towards the diagnosis of Van Wyk-Grumbach syndrome as other causes of precocious puberty usually are associated with accelerated growth velocity and advanced bone age.

Based on a literature search, Van Wyk-Grumbach syndrome has seldom been described in patients with trisomy 21 [11]. Although the prevalence of hypothyroidism in trisomy 21 is high, presentation in the form of Van Wyck-Grumbach syndrome is quite rare. Patients with Trisomy 21 may have clinical features that overlap with hypothyroidism such as short stature, developmental delay, hypotonia, and dry skin. A change is baseline clinical status in a patient with trisomy 21 such as decreased growth velocity, increased fatigue, or decreased tone should alert the physician about the possibility of underlying hypothyroidism. This case underscores the importance of screening for hypothyroidism in patients with trisomy 21 [12] and in children presenting with precocious puberty. Early recognition of the clinical presentation of Van Wyck-Grumbach syndrome and treatment with Levothyroxine can help avoid unnecessary investigations, patient anxiety and treatment.

## Data Availability

Data sharing is not applicable to this article as no datasets were generated or analyzed during the current study.

## Abbreviations

AFP: Alpha-fetoprotein
CA-125: Cancer antigen 125
FSH: Follicle stimulating hormone
LDH: Lactate dehydrogenase
LH: Luteinizing hormone
TSH: Thyroid stimulating hormone
